# Pathological assessments for the presence of hexanucleotide repeat expansions in *C9ORF72* in Alzheimer’s disease

**DOI:** 10.1186/2051-5960-1-50

**Published:** 2013-08-12

**Authors:** Yvonne S Davidson, Andrew C Robinson, Julie S Snowden, David MA Mann

**Affiliations:** 1 Clinical and Cognitive Sciences Research Group, Institute of Brain, Behaviour and Mental Health, Faculty of Medical and Human Sciences, University of Manchester, Salford Royal Foundation NHS Trust, Salford M6 8HD, UK

**Keywords:** Alzheimer’s disease, Genetics, C9ORF72

## Abstract

**Background:**

We have sought histological evidence, using TDP-43 and p62 immunohistochemistry, for the presence of expansions in *C9ORF72* among 200 patients with pathologically confirmed AD.

**Results:**

We noted TDP-43 pathological changes in hippocampus and temporal cortex in 45 (22.5%) of these patients, but did not detect any cases where p62 positive changes in hippocampus and cerebellum, considered pathognomic for *C9ORF72* expansions, were present.

**Conclusion:**

We conclude that expansions in *C9ORF72* associated with AD are a rare occurrence, and in those instances in the literature where these have been reported, the presence of AD may in fact be coincidental and unrelated to the expansion.

## Background

Frontotemporal Lobar Degeneration (FTLD) is a clinical, pathological and genetically heterogeneous condition. The major clinical syndromes principally involve personality and behavioural change (behavioural variant frontotemporal dementia, or bvFTD) or language alterations of a fluent (semantic dementia) or non-fluent (agrammatic aphasia) nature [1]. All three syndromes can be accompanied by Motor Neurone Disease (MND), though bvFTD and MND is the most common combination. Histologically, about half of cases have tau-based pathology, half have TDP-43-based pathology, and about 5% have FUS-based pathology [2]. Importantly, around 40% of cases have a strong family history of similar disease, irrespective of clinical or histological subtype, and associate with variations in three major genes – mutations in tau (*MAPT*) [3] and progranulin (*GRN*) [4,5], or a large hexanucleotide (GGGGCC) expansion in *C9ORF72*[6-8].

The expansion in *C9ORF72* occurs in the first intron or the promoter region of the gene, depending upon the transcript isoform in question, and can number up to as many as 1500 repeats [6-8]. The expansion is found in about one in every twelve patients with FTLD or MND, making it the most common genetic causes of both these disorders [6-8]. Pathologically, most cases of FTLD bearing the expansion [6-12], like many non-mutational cases of FTLD [2], show inclusion bodies within neurones (NCI) and glial cells of the cerebral cortex and hippocampus that contain the nuclear transcription factor, TDP-43, and are said bear a TDP-43 histological subtype termed FTLD-TDP type B (according to Mackenzie classification [13]), compatible with a clinical diagnosis of FTD and MND. Others, however, show a TDP-43 histological type characterised by the presence of many short neurites (DN) along with NCI within the outer layers of the cerebral cortex, and are termed FTLD-TDP type A [13]. Nonetheless, irrespective of TDP-43 histological type, all patients with FTLD (and indeed those with MND) reported to bear expansion in *C9ORF72* also show a unique pathology within the dentate gyrus granule cells and CA4 pyramidal cells of the hippocampus [9] and granule cells of the cerebellum [9,10] characterised by NCI that are TDP-43 negative, but immunoreactive to p62 protein [6-12].

Interestingly, expansions have been reported to occur within patients with other neurodegenerative disorders, particularly Alzheimer’s disease (AD) ( [14-18] but see [19,20]). However, in many of these individuals where an expansion has been reported [14-18], there has been no pathological confirmation of disease histology, and therefore the underlying condition in such cases could still be FTLD, though presenting in an atypical way. Nonetheless, there do still appear to be some cases of clinically relevant and pathologically confirmed AD bearing expansions in *C9ORF72*[16,17].

About 25-45% cases of AD also show (secondary) TDP-43 pathological changes within the amygdala, hippocampus, and sometimes also in neocortical regions [21-24], the proportion of ‘positive’ cases seemingly being dependent upon how extensively the brain is sampled [21] Cases are frequently encountered where TDP-43 pathological changes are confined to the amygdala, and consequently such ‘incidental’ cases may be considered negative if this region is not examined [21]. However, when the neocortex and hippocampus are also involved, the histological pattern usually resembles that of FTLD-TDP type A [21,22]. In those cases of pathologically confirmed AD where expansions are present [16,17] no TDP-43 or p62 immunostaining had apparently been performed. It is therefore possible that cases of AD bearing expansions, like those with FTLD or MND [6-12], should show p62 changes in the hippocampus and cerebellum, as well as a widespread TDP-43 proteinopathy.

Given that p62 changes in the hippocampus and cerebellum might be pathognomic for expansions in *C9ORF72*[9,11], we immunostained sections of temporal cortex (with hippocampus) and cerebellum in 200 pathologically confirmed cases of AD in order to ascertain to what extent an expansion in *C9ORF72* might be present in AD, particularly in those cases with (extensive) TDP-43 pathology, since it is possible that such cases, in the context of a clinical picture resembling AD, might be ones with undisclosed/unrecognised FTLD.

## Methods

Brain tissues were available in the Manchester Brain Bank from a series of 200 patients with pathologically confirmed AD (96 with early onset AD, 104 with late (ie after 65 years) onset AD. All brains had been obtained with full ethical permission following consent by the next of kin. Paraffin sections were cut (at a thickness of 6 μm) from formalin fixed blocks of temporal cortex (with hippocampus) and cerebellar cortex and immunostained by routine methods, involving pressure cooking for antigen retrieval, for TDP-43 [22], and p62-lck ligand (rabbit polyclonal antibody (B D Biosciences, Oxford, UK) 1:100), employing a standard ABC Elite kit (Vector, Burlingame, CA, USA) with DAB as chromagen [22].

Immunostained sections were assessed for presence of TDP-43 pathological changes within the hippocampus dentate gyrus, the entorhinal cortex/fusiform gyrus and the inferior and middle temporal gyri [22]. P62 pathological changes were sought within the dentate gyrus and CA2/3/4 regions of the hippocampus, and within the granule cell layer of the cerebellum, as described previously by Al-Sarraj et al. [9], and Boxer et al. [10], respectively. Positive cases were defined where either TDP-43 positive, or p62 positive, TDP-43 negative NCI, within either the cerebellum or hippocampus could be clearly seen under low power objective (X20) and the majority of high power fields (x40) contained at least 2 NCI. Negative cases were either completely devoid of TDP-43 or p62 immunostaining, or showed only small amounts of apparently extracellular and ‘extraneous’ p62 positive particulate material in occasional high power fields.

## Results and discussion

Trading on the ‘tightly coupled’ association between p62 pathological changes in the hippocampus and cerebellum and the possession of hexanucleotide repeat in *C9ORF72* in FTLD [6-12], we sought to determine, from a pathological basis, whether such expansions might occur in patients with histopathologically verified AD. Consequently, we screened 200 cases of AD for widespread TDP-43 pathological changes in hippocampus and temporal lobe, and for the presence of p62 positive NCI within hippocampus (dentate gyrus, CA2/3/4 cells) and cerebellum (granule cells). Forty five (22.5%) of the 200 cases of AD showed TDP-43 pathology mostly resembling FTLD-TDP type A histology [13], within the dentate gyrus granule cells and entorhinal cortex/fusiform gyrus, and within layer II pyramidal cells of inferior and middle temporal gyri (as described previously by us [22] and others [21,23,24]). However, no p62 immunoreactive NCI were seen within pyramidal cells of areas CA2/3/4, or within dentate gyrus granule cells, of the hippocampus [9], or within granule cells of the cerebellum [10], in any of these 45 cases, or in any of those cases of AD not showing TDP-43 pathology. Hence, we conclude that, even in the absence of formal genetic analysis, none of the 200 cases of AD examined here would likely be carriers of *C9ORF72* expansion.

All previous studies have been based either, exclusively on cohorts of patients with clinically diagnosed AD, or on cohorts with clinical AD but with limited pathological investigations in some expansion bearing, or non-bearing, individuals [14-20] (see Table 1). Collectively, these 7 studies have investigated 5511 patients with clinically diagnosed AD. A total of 38 patients were found to bear expansions in *C9ORF72*. Of these, of further clinical review, 24 were still considered to have AD, and this was confirmed on pathology in 4 instances. Of the other 14 patients, 12 were considered to have FTLD on further review of the clinical notes, and two had this diagnosis confirmed on pathology. Hence, of all the 38 expansion carriers disclosed in these studies, only 4 could be said to have definite AD. Therefore, in the absence of pathological confirmation of disease, it remains possible that many of the remaining carriers of expansions in *C9ORF72* with a clinical presentation compatible with AD could actually represent phenocopies of FTLD. Nonetheless, it does appear that there are still a few cases with an expansion in *C9ORF72* where AD has indeed been confirmed pathologically either in the index patient, or within other members of the families involved [16,17].

**Table 1:** **Details of the 7 previous studies investigating the presence of a hexanucleotide expansion in** ***C9ORF72*** **in patients with Alzheimer’s disease**

**Study**	**Total number of cases investigated with clinical AD**	**Number of cases with expansion in *C9ORF72***	**Number of cases with expansion in *C9ORF72 *** **with AD confirmed by pathology (or on further clinical review)**	**Number of cases with expansion in *C9ORF72 *** **but with other disorders confirmed by pathology (or on clinical review)**
[14]	904	11	0 (0)	0 (11)
[15]	1217	5	0 (5)	0 (0)
[16]	872	5	1 (4)	0 (0)
[17]	1184	11	3 (6)	1 (1)
[18]	342	6	0 (5)	1 (0)
[19]	568	0	0	0
[20]	424	0	0 (0)	0 (0)
Total	5511	38	4 (20)	2 (12)

However, it is noted that, in these latter studies [16,17], expansion bearing cases had not had a full histopathological work up, including p62 and TDP-43 immunohistochemistry, in order either to confirm the expansion pathologically, or to rule out coincident FTLD or other neurodegeneration. Moreover, in at least 2/4 such cases, onset of disease had occurred after 70 years of age [17], with both individuals bearing Apolipoprotein E ϵ4 allele, one being homozygous for this [17]. We have previously shown that the presence of Alzheimer-type pathology, which may be sufficient in some instances to meet pathological criteria for AD, can occur in elderly cases of FTLD, especially those bearing Apolipoprotein E ϵ4 allele [25], and particularly so within unaffected regions of the posterior hemisphere [26]. It is therefore possible that the occurrence of AD pathology in expansion bearers in the aforementioned studies [16,17] is entirely coincidental, and unrelated to the expansion. Indeed, we have also shown that elderly subjects with FTLD commonly present with an AD-like syndrome [27]. Hence, it remains possible that in such individuals with AD pathology, the expansion in *C9ORF72* might in fact may be responsible for directing an FTLD type of process, which has not be disclosed, since apparently in none of these studies had expansion carriers been investigated for these kinds of histological changes.

## Conclusions

In conclusion, in the present study, we sought histological evidence for the presence of expansions in *C9ORF72* among 200 patients with pathologically confirmed AD, but did not detect any cases where relevant tissue changes were present. We conclude that expansions in *C9ORF72* are a rare occurrence in AD, and in instances where these do occur, the presence of AD may be coincidental and unrelated to the expansion. Further investigations on such cases to exclude the presence of an underlying FTLD process driven by the expansion appear warranted.

## Competing interests

The authors declare that they have no competing interests.

## Authors’ contributions

YD performed all immunohistochemistry. AR provided technical assistance. JS helped with clinical characterisation of patients; DM performed study design, data and slide analysis and wrote the paper. All authors read and approved the final manuscript.
